# Perspectives of stakeholders regarding the value of maternal and newborn health interventions and practices supported by UNICEF and other partners in the West Nile region of Uganda: a qualitative study

**DOI:** 10.1186/s12913-023-09480-x

**Published:** 2023-05-11

**Authors:** Simon Muhumuza, Xavier Nsabagasani, Cinderella Ngonzi Muhangi, Grace Latigi, Patricia Pirio, Chimwemwe Msukwa, Fabian Mwanyumba, Fatima Gohar, Tedbabe Degefie Hailegebriel, Atnafu Getachew Asfaw, Anne-Marie Bergh

**Affiliations:** 1 School of Public Health, Makerere University, P.O. Box 7072, Kampala, Uganda; 2 UNICEF, PO Box 2381, Kampala, Uganda; 3 UNICEF Eastern and Southern African Regional Office, P.O. Box 44145-00100, Nairobi, Kenya; 4 UNICEF Headquarters, New York, NY 10017 USA; 5 Research Centre for Maternal, Fetal, Newborn and Child Health Care Strategies, Faculty of Health Sciences, University of Pretoria, Private Bag X323, Gezina, 0031 South Africa

**Keywords:** Maternal health, Newborn health, Programme implementation, Health-system levels, Health-systems building blocks, Health-systems strengthening, Uganda

## Abstract

**Introduction:**

Uganda has high maternal, neonatal, and under-five mortality rates. This study documents stakeholder perspectives on best practices in a maternal and newborn health (MNH) quality-improvement programme implemented in the West Nile region of Uganda to improve delivery and utilisation of MNH services.

**Methods:**

This exploratory cross-sectional qualitative study, conducted at the end of 2021, captured the perspectives of stakeholders representing the different levels of the healthcare system. Data were collected in four districts through: interviews with key informants working at all levels of the health system; focus group discussions with parents and caretakers and with community health workers; and interviews with individual community members whose lives had been impacted by the MNH programme. The initial content analysis was followed by a deductive synthesis pitched according to the different levels of the health system and the health-systems building blocks.

**Results:**

The findings are summarised according to the health-systems building blocks and an account is given of three of the interventions most valued by participants: (1) data use for evidence-based decision making (with regard to human resources, essential reproductive health commodities, and financing); (2) establishment of special newborn care units and high-dependency maternity units at district hospitals and training of the health workforce (also with reference to other infrastructural improvements such as the provision of water, sanitation and hygiene facilities at health facilities); and (3) community referral of pregnant women through a commercial motorcycle voucher referral system.

**Conclusion:**

The MNH programme in the West Nile region adopted a holistic and system-wide approach to addressing the key bottlenecks in the planning, delivery, and monitoring of quality MNH services. There was general stakeholder appreciation across the board that the interventions had the potential to improve quality of care and newborn and maternal health outcomes. However, as the funding was largely donor-driven, questions about government ownership and sustainability in the context of limited resources remain.

**Supplementary Information:**

The online version contains supplementary material available at 10.1186/s12913-023-09480-x.

## Introduction

The reduction of maternal, newborn and under-five mortality remains a challenge in sub-Saharan Africa and the coronavirus pandemic may have stalled or reversed recent progress [1]. Despite support for the three overarching objectives of ‘survive, thrive and transform’ of the 2016–2030 Global Strategy for Women’s, Children’s and Adolescents’ Health [2], many countries are not on track to reach the 2030 targets for Sustainable Development Goal 3, namely reducing the global maternal mortality ratio (MMR) to less than 70 per 100,000 live births, the neonatal mortality rate to 12 per 1,000 live births and the under-5 mortality rate to 25 per 1,000 live births [3]. The government of Uganda’s Sharpened Plan for Reproductive, Maternal, Newborn, Child and Adolescent Health (RMNCAH) 2016/2017–2019/2020 focused on addressing underlying constraints and accelerating progress in maternal and child health within the context of the Sustainable Development Goals and the global strategy [4]. This plan outlined the priority interventions for reducing maternal, newborn, and child mortality and described high-impact interventions for addressing the continuum of care that extends through adolescence, pregnancy, childbirth, infancy and childhood. The revised RMNCAH Sharpened Plan 2020/21–2025/6 sets ambitious targets for 2025 with regard to reducing the institutional MMR from 311 to 211 per 100,000 live births and reducing the NMR, which has stagnated at 27 per 1,000 live births for over 20 years, to 12 per 1,000 live births [5].

### Maternal and newborn health in the West Nile region, Uganda

The West Nile region in the northwest corner of Uganda shares borders with the Democratic Republic of the Congo and South Sudan. It also hosts more than half of the 1.5 million refugees in the country [6]. The region is classified among the lagging regions of Uganda, hardest hit by food insecurity, with poverty significantly above the national average [7], and conditions that strongly affect maternal and newborn health (MNH) outcomes, in terms of both general health status and capacity to access services. Historically, dismal MNH indicators have been common in the region [8–10].

In 2018, the West Nile region embarked on the delivery of an integrated MNH quality-improvement package with substantial investments in high-impact supply-side and demand-side interventions to address key barriers to the availability and utilisation of quality MNH services in all 12 districts. The programme targeted the three delays leading to maternal and newborn deaths, namely delays in the decision to seek care, in arrival at an appropriate health facility, and in the provision of immediate and adequate care [11]. The focus was both on strengthening all levels of the health system and its building blocks to enable rapid response to the needs of mothers and newborns and on increasing community awareness, demand, acceptance, and utilisation of available MNH and nutrition services. Several development partners, donors, and agencies collaborated with the Ministry of Health and district health teams to support different components of the programme. A substantial number of the initiatives were supported by the United Nations Children’s Fund (UNICEF) through implementing partners such as the Association of Volunteers in International Service (AVSI) Foundation. Other partners included the Johns Hopkins Program for International Education in Gynecology and Obstetrics (Jhpiego), the United Nations Population Fund (UNFPA), the International Rescue Committee (IRC) and Marie Stopes Uganda (MSU).

Because of multiple-partner involvement and the comprehensive health-systems strengthening (HSS) approach, it is not possible to measure the impact of individual interventions or activities on the improvement of the MNH programme and maternal and newborn outcomes in the West Nile region. The aim of this study was therefore to answer the following question: What are the perspectives of stakeholders at the various levels of the healthcare system regarding what they consider to be the most valued MNH interventions and practices supported by UNICEF and other partners in the West Nile region of Uganda?

## Methodology

### Study design

The choice of study design was guided by available budget and UNICEF’s desire to get more in-depth feedback from stakeholders at all levels of the health care system on what they perceived to be the most valuable MNH interventions in terms of improving MNH outcomes. An exploratory cross-sectional design using qualitative methods of data collection was adopted. The data collection techniques were key informant interviews, focus group discussions and in-depth interviews. For data analysis, two widely implemented frameworks – the WHO’s health-systems building blocks [12] and the different levels of the health system [13] – were selected as the lenses for categorising the interventions perceived to be of value and the effect the MNH programme had on people’s lives. The analysis also considered the supply- and demand-side shortcomings, challenges and barriers associated with these interventions.

### Study setting

To attain maximum diversity, four of the 12 districts in the West Nile Region were purposively sampled for inclusion in the study. Two high-performing (D2 and D3) and two poorly performing (D1 and D4) districts were selected, based on their ranking in the national district league table of 2017/8 [14]. The two poorly performing districts were classified as high-burden districts with regard to neonatal and under-five mortality and unmet family planning needs and the better performing districts were rated as middle-burden districts [4]. The four districts constitute 50% of the total population of the West Nile region [15], host 60% of the refugees in the region [6] and are characterised by poor access to and low quality of health care services. In 2017/18, the four districts registered some of the poorest MNH indicators in the region, including low antenatal care attendance of 19.4% in the first trimester of pregnancy and high in-facility stillbirths at 87 per 1,000 births [14].

### Study population and sampling

The study participants were drawn from the national, regional, district, health facility and community levels and comprised MNH programme managers, district health managers, health facility managers and healthcare providers, community health workers and community members. They were purposively selected based on their position, knowledge, experience of and/or involvement with the MNH programme. National and regional participants included UNICEF country and zonal MNH technical officers, respectively. They were health professionals with a background in medicine, nursing and public health.

Participants at the district level were members of the district health team and included district health managers and biostatisticians, who are the custodians of district-health data. Through snowball sampling, the district health managers helped the investigators to recruit eligible health-facility managers and providers. They were drawn from four hospitals (3 public, 1 private-not-for-profit) and three health centres that were beneficiaries of UNICEF partner-driven support.

Health-facility participants adopted the same approach to help identify and recruit community-based informants. Community health workers, commonly known as village health teams (VHTs) were purposively sampled from the communities served by health centres (*n* = 9) that participated in the MNH programme. Parents and caretakers of children under one year of age and other individuals with unique stories about how the MNH programme impacted their lives were identified by VHTs through snowball sampling.

### Recruitment and data collection

Data collection took place in November and December 2021. A total of 99 participants took part in the different interview types, 33 males and 66 females. Table 1 contains a more detailed summary of the participants.

**Table 1:** Summary of study participants

**Study participants**	**Inclusion criteria**	**n**	**Gender**
**Key informant interviews**			
Health facilities	Managers and care providers in health facilities where the MNH programme was implemented	7	6 M; 1 F
District health offices	District health officials involved in the design, implementation and monitoring of the MNH programme	7	3 M; 4 F
Zonal and national level	• Knowledge and understanding of the MNH programme • Regional/zonal level: Officials responsible for planning, supervising, monitoring and reporting on the MNH programme at this level • National level: MNH technical officers from UNICEF country office	4	2 M; 2 F
**Subtotal**		**18**	**11 M; 7 F**
**Focus group discussions**			
Families (mothers)^a^ (4 FGDs)	Parents (mothers/caretakers) of children <1 year of age accessing care at facilities with model maternity and neonatal units established with support from the MNH programme	36	36 F
Village health team members (5 FGDs)	Trained community health workers involved in the MNH programme	33	16 M; 17 F
**Subtotal**		**69**	**16 M; 53 F**
**Individual interviews**			
Women/mothers	Individuals or groups of people with stories of interest relating to how their lives (or the lives other people in their social circles) were impacted by the MNH programme	4	4 F
Village health team members		4	2 M; 2 F
Boda-boda riders		4	4 M
**Subtotal**		**12**	**6 M; 6 F**
**TOTAL participants**		**99**	**33 M; 66 F**

^a^Male involvement in reproductive care is still very limited in the West Nile region and no males were available for inclusion on the days of data collection

*FGD* focus group discussion, *M* male, *F* female

**Fig. 1:**
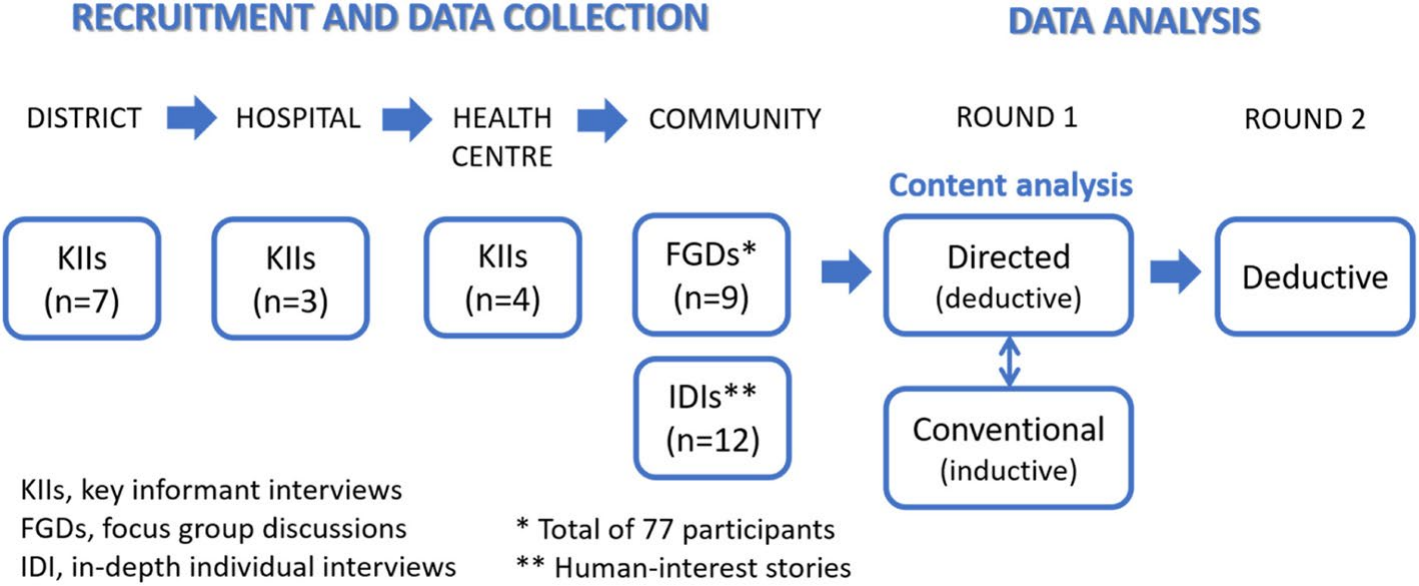
Data collection and analysis methods

Data collection followed a specific sequence in each district (see Fig. 1). District offices were targeted first to obtain both administrative clearance for the study and permission to conduct interviews with district-level participants. This was followed by interviews held at the hospital, health centres and in the community.

Each data collection method had its own topic guide with open-ended questions, which were informed by information gleaned from an informal review of relevant government and development partner documents pertaining to the MNH programme in the West Nile region. The guides for community participants were developed in English by the investigators and later translated into the local dialects of Lugbara, Alur, and Madi. They were subsequently reviewed by research assistants fluent in the relevant local language.

The first three authors held key informant interviews (KIIs) with managers and healthcare providers from all levels of the health system who were considered to be information rich – zonal and national officials (ZN), district health officials (DO), health-facility (HF) participants (including medical superintendents and those in charge of health centres).

Research assistants fluent in the local language conducted focus group discussions (FGDs) and in-depth individual interviews (IDIs) with community members. The eight assistants (4 males, 4 females) resided in the study districts; all held at least a bachelor’s degree in humanities and some had previous experience in interviewing. They all received thorough orientation and training in the study protocol, the technical interventions and interviewing techniques. In order to elicit more general views of community members on the MNH interventions at community level, separate FGDs with 6-8 participants each were held with each community category (community health workers and parents/caregivers), two discussions per district. The focus of the IDIs was to record ‘human-interest stories’ from individuals who had benefited from specific MNH interventions, such as efforts to promote delivery at health facilities. In each district, a mother, a VHT member and a commercial motorcyclist (a boda-boda rider) (BB) were interviewed to cover perspectives on some of the most important demand-side delays addressed by the interventions.

Interviews lasted between 40 and 60 minutes and all interviews were audio-recorded with permission. KIIs were held in the privacy of regional, district and health-facility offices and other convenient venues like hotel premises. Private places were designated in the communities for FGDs and IDIs. Due to the short timeframe and the fact that interviews took place concurrently in different districts, data saturation could not easily be monitored. The subsequent data analyses revealed recurrent information from the different districts and at the different levels.

### Data management and analysis

All the audio-recorded interviews and FGDs were transcribed verbatim and those conducted in the local languages were translated into English. Transcripts were saved electronically with unique ID codes. Audio-recordings and documents were securely kept in the first author’s office.

Data analysis consisted of a combination of inductive and deductive analysis (Fig. 1). The first round of analysis was guided by two different approaches to analysing the content of 38 transcripts. Researchers familiarised themselves with the data, generated initial codes and searched for themes [16]. The first three authors used a directed approach [17] to content analysis and worked together in an iterative process of manual analysis of the data, organising relevant quotes according to specific categories or codes, and developing a coding scheme related to the different health-system levels. The last author performed a conventional content analysis [17] to define codes during data analysis with a view to confirming the feasibility of the interpretation of the first three authors. The supplemental file contains an example of the nodes created from the KIIs using NVivo9 software. The categories and topics that emerged from the two analyses were discussed, compared and combined.

In the second round of analysis, we used the health-systems building blocks [12] and the health-system levels [13] deductively to synthesise the valued interventions emerging from the first round of analysis. This was aimed at aligning our analysis with the conceptualisation of the interventions that were included in the MNH quality-improvement programme documents.

### Rigour

Various measures were put in place to enhance the rigour of the study, using Nowell *et al.*’s pointers for establishing trustworthiness [16]. Credibility is demonstrated by the regular discussions between the researchers from the inception of the study to the report-writing stage and by the audit trail of field notes and coding decisions that was kept. Although the study was funded by UNICEF and its officials contributed to the study design, none of them was involved in the data collection and analysis or the interpretation of findings. Drafts of the study report were circulated among national, zonal and district-level managers in the West Nile region and the study districts for comments. As some of these individuals were key informants in the study, this could be considered a form of member checking. UNICEF also gave feedback to stakeholders at all levels through a PowerPoint presentation and by sharing the final report. With regard to the study itself, researchers from two institutions analysed the data independently, and verbatim quotations from participants are included in this paper. Some of the findings may be transferable to other districts in Uganda with similar populations and contexts. Triangulation, achieved through the inclusion of different categories of informants (district managers, service providers, users of the services and agents in service access) and different data collection methods, demonstrates dependability. Confirmability in study design and data analysis was enhanced by the combination of independent researchers from different institutions and programme staff with diverse disciplinary backgrounds.

### Ethical considerations

Ethical clearance was obtained from the Makerere University Higher Degrees Research and Ethics Committee (SPH-2021-162). Permission for the study was obtained from the respective district health offices and managers of health facilities. Written informed consent was obtained from all participants. The data collectors signed confidentiality agreements.

## Results

The MNH interventions in the West Nile region should be considered against the macro-level policies and strategic directions of the national development plan. Health-system interventions are endorsed and supervised by the Ministry of Health. Partners saw their role as supporting these policies and strategies, but following a district-needs, HSS approach, to supplement government contributions – ‘they [the partners] keep saying, identify your gap and tell us what you want’ (KII HF D3). Health-system stakeholders perceived all the interventions as equally important in a holistic approach – ‘you cannot really choose because they are all targeting different … [but] not competing priorities’ (KII HF D3).

The findings are divided into four broad topics. Firstly, the MNH interventions and activities valued by stakeholders are described according to health-systems building blocks. This is followed by a more detailed account of three of the most valued MNH interventions: using data to improve the quality of MNH care (district and facility level); upgrading neonatal and maternity units and training the health workforce (facility level); and the boda-boda motorcyclist referral system (community level).

### MNH interventions and activities valued by stakeholders

Table 2 summarises the main interventions in the West Nile MNH programme as well as the challenges mentioned by stakeholders, organised according to the WHO’s health-systems building blocks [12]. Some of the interventions were more general, such as the provision of water, sanitation and hygiene (WASH) facilities and the use of data to improve services, whereas others, such as the upgrading of maternity and neonatal units, were specifically focused on improving the quality of MNH care. Interventions considered pivotal were those pertaining to capacity building of managers and healthcare providers and to access to and delivery of safe, quality services.

**Table 2:** MNH interventions and activities valued by stakeholders, according to the health-systems building blocks

**Building block**	**Intervention**	**Description**	**Purpose**	**Challenges and gaps**
**Service delivery**	Provision of WASH (in selected facilities)	• Connecting borehole water to health facilities, including rehabilitation of old, non-functional water systems • Establishing related infrastructure (e.g. tanks, toilets, taps, sinks, bathrooms) • Use of water: washing hands, linen, and clothes; cleaning; cooking food • COVID-19: personal protective wear; coveralls, gloves, soap and sanitisers	• Prevent and reduce infections • Increase service utilisation	• WASH facilities largely donor funded • No regular allocated budget for maintenance – sustainability uncertain • WASH facilities not exclusively for MNH services • Sanitation infrastructure still inadequate for the over-crowded health units • Waste treatment needs attention
	Renovation of (selected) health facilities to provide for a higher level of care	Establishment of well-equipped units for around-the-clock care: • Neonatal intensive care – provision for sick and small newborns • High-dependency units – high-risk women with obstetric complications	• Improve quality of care: round-the-clock personalised care by skilled providers • Reduce maternal and neonatal mortality	**•** Outdated staffing structure – no additional staff hires **•** Unreliable power supply affects operation of equipment and effectiveness of these care units • Increased maintenance and utility costs – increase in power demand
	Improved referral and emergency transport systems	Car ambulances between health facilities	Reduce delays in reaching health facilities in emergency situations	• Occasional lack of fuel • Frequent breakdowns • No provision for their maintenance
		Boda-boda transport with motorcycles from community to health facility	Reduce delays in reaching health facilities in emergency situations	**•** Delayed payment of vouchers – motorcycle operators demoralised • Type of motorcycles not appropriate for carrying high-risk pregnant women with danger signs • Impassable roads during extreme wet and dry weather conditions • Poor road infrastructure and the long distances to health facilities affect the operations of ordinary motorcycles
**Health information systems and mobile health technology**	Use of data for monitoring MNH services	**•** Real-time monitoring and action performance (RTMAP) used in support of bottleneck analysis **•** Use of scorecard methodology **•** Maternal and perinatal death surveillance and response (MPDSR) **•** Periodic data quality assessments **•** Quarterly performance reviews **•** Annual health-facility quality assurance programme assessment **•** Annual client satisfaction surveys **•** Annual district health-system capacity assessments	• Improve performance of district • Inform decision making, planning and budgeting	• Limited ability of district staff to conduct data analysis • Multiple and parallel data collection and reporting systems • Insufficient infrastructure to support electronic data management, reporting, and use (e.g. computers, internet connectivity and unreliable power)
	Functionalisation of the integrated human resource information system (iHRIS)	• Updating of district staff lists	• Assist with human resource planning	• The iHRIS not updated regularly
	FamilyConnect	Targeted lifecycle-based messages • Sent via SMS to pregnant women, new mothers, heads of households, male partners, and caregivers • Information on what recipients can do to keep themselves and their babies in good health	• Improve uptake of MNH services	**•** Only rolled out in two of the study districts **•** Low mobile phone coverage in the region **•** Limited telephone network in some areas • Usage influenced by negative socio-cultural beliefs about pregnancy, antenatal and postnatal care; gender-based violence and widespread poverty
**Health workforce**	Training and capacity building across all levels of health care	District: training of DHTs and sub-district staff on ⁃ bottleneck analysis ⁃ the RMNCAH scorecard ⁃ MPDSR Health-facility level: ⁃ ANC, BEmONC, CEmONC, MPDSR, essential newborn care, key family care practices (KFCPs), use of BABIES matrix, ASRH, etc. ⁃ Capacity expansion of HCs III to conduct normal deliveries and only refer those at risk ⁃ Specialised training for staff in neonatal care and obstetric emergencies • Training of community health workers in family care practices	• Improve skills that lead to improved quality of care • Reduce congestion at high-volume facilities • Reduce referrals to regional hospitals	**•** Gaps in HR capacity (required cadres and skills) **•** Shortage of critical staff for new units established **•** Newly qualified midwives not well oriented on the key practices
	Post-training support	Onsite coaching, mentorships and supportive supervision	• Further improve skills • Identify bottlenecks in service delivery	• Inadequate funding for fieldwork
	Staff recruitment (especially critical staff)	• UNICEF supported the recruitment process (advertising for positions and interviews) • Advocacy for a wage bill increment • Ongoing lobbying for different funding streams for human resources for health (e.g., results-based financing)	Reduce shortage of critical staff for MNH service provision	• Underfunding of human resources – inadequate wage • No reforms in staffing norms • Challenges in government’s recruitment processes • Staffing levels not commensurate with workload • Shortages of critical staff, particularly medical officers, midwives and nurses
**Medical products, vaccines and technologies (Supply chain)**	Procurement of equipment	• Facilities indicate to partners what they need and partners procure and deliver equipment • Partners follow up about the functionalisation and use of the equipment	Ensure appropriate usage of donations	• Equipment not always according to local standards – could affect functionality
	Reproductive health commodities	Partners supplemented various commodities and medicines when health facilities ran short	Save lives and treat conditions	• Not all gaps filled • Lack of sufficient medications could lead to death
	Establishment of blood bank	• UNICEF supported the refurbishment of the blood bank at general hospital in District 3 and provided additional power regulators • Uganda Blood Transfusion Services and Ministry of Health engaged to accredit these blood transfusion services • Procurement and distribution of equipment to lower level health facilities in case of need	Save maternal lives	• A blood bank not available in every district • Districts still rely on the regional referral hospital for blood transfusion services • Cost to collect blood from the regional hospital • Availability of blood
**Health-system financing**	Financing partners	Financing of the various health-systems building blocks	Improve service delivery and quality of care	• Inadequate finances to address all gaps • Delay in transfer of funds to districts • Lack of sustainability strategies for big investments
	Leverage of additional resources through the government (PHC) and performance-based financing (PBF)	Funding for performance measured against indicators at facility level	Strengthen districts’ ability to provide services	• Lack of financing for coordination and monitoring of interventions • Weak coordination of funding streams • No specific budget line for maternal and newborn health
**Leadership and governance**	Coordination of integrated health plans and MNH programmes	• Mapping of partners in the districts • Development of one-health plans and budgets • Planning use of the bottleneck analysis approach	• Minimise duplication of efforts • Enhance effective and efficient service delivery	• Inadequate governance and leadership skills among health managers • Lack of accountability
	Establishing functional committees	• MPDSR committees • Health unit management committees	• Monitor service delivery and planning	• Inadequate funding to support the committee functions

*ANC* antenatal care, *ASRH* adolescent sexual and reproductive health, *BEmONC* basic emergency obstetrics and newborn care, *CEmONC* comprehensive emergency obstetrics and newborn care, *DHTs* district health teams, *HC* health centre, *HR* human resources, *KFCPs* key family care practices, *MNCH* maternal, newborn and child health, *MNH* maternal and newborn health, *MPDSR* maternal and perinatal death surveillance and review, *PHC* primary health care, *PNC* postnatal care, *RMNCAH* reproductive, maternal, newborn, child and adolescent health, *SMS* short message services, *VHT* village health team, *WASH* water, sanitation and hygiene

Coverage of a wide range of interventions was made possible by heavy investment by UNICEF through its development partners. The outstanding challenge is for the district local governments in the region to provide oversight and, in time, take over, scale up and sustain these expensive interventions – ‘at the district [level], local government [should] have [a] certain level of commitment’ (KII ZN). A number of informants identified the two main challenges for healthcare facilities:

‘If finances are there and in the right hands, … the two biggest things for a healthcare facility [are] human resource[s], and then of course, … medicines and supplies. We need … good governance’ [our emphasis] (KII HF D3).

### Use of data to improve the quality of maternal and newborn care

At the district, zonal and national levels, participants referred to the partner-supported training activities for district and hospital administrators in planning, management and budgeting to enable them to do evidence-based planning using bottleneck analysis and real-time monitoring and action performance (RTMAP). This is how a district biostatistician explained the way they used data:

‘All the interventions that we implement within the district health sector should be based on evidence-targeted interventions, … and this evidence is actually the data. … So whatever activity we are doing, the [annual] work plan that we generate … is arrived at by analysing data. For example, we are not going to say we are going to do community dialogues if the choice of that activity was not informed by data. So, we always do things according to data. Every activity is backed by evidence. … And it is the direction that even now the Ministry wants. We are not going to say we are going to do this training when we have done our analysis and there's no indication that there was need for training, because … my work is to analyse the data and make the presentation and then we analyse it together. And then we get the root cause of the problem from that data. And that is what informs our work plan, our programming. So, we're not going to tolerate just any activity from any partner, when data has shown that there is no problem there’ (KII DO D2).

Data were also used to identify and analyse the factors contributing to maternal and newborn health outcomes. Reference was made to support for quarterly maternal and perinatal death surveillance and response (MPDSR) meetings that provided crucial data for the district health team’s monitoring of district performance and identification of bottlenecks related to the three delays in care.

One official reflected on HSS based on reliable data as a process that takes time to establish:

‘Many of the interventions happening right now really seem to be new interventions with the results. But it needs time for the district and the local government team to be well versed with data and therefore implement with confidence’ (KII ZN).

Data-based performance included review across the different health-systems building blocks – ‘Through the regular performance review meetings … we jointly generate solutions for improving performance’ (KII DO D3). Three specific building blocks were pertinent in the stakeholder perspectives: human resources; essential reproductive health commodities; and financing.

#### Human resources

With regard to human resources, reviews highlighted the lack of adequate financial resources to employ sufficient numbers of staff and to conduct MNH capacity building and training activities for which support had been negotiated with development partners. For example, UNICEF supported the districts with ‘the placement, including payment of salary, of the critical staff’ (KII ZN) and complemented the efforts of the district service commissions to recruit and second critical staff cadres in circumstances where there were no funds for recruitment and where the wage bill was not fully utilised by the districts – ‘This staff was initially supported by UNICEF directly, but later on, the district absorbed them’ (KII DO D2).

#### Essential reproductive health commodities

The performance reviews also considered the availability of essential commodities and proposed solutions for delays in deliveries of commodities from the national medical stores and for challenges in ordering appropriately and on time. Participants at various levels of the health system referred to the support they had received from partners to adequately prioritise reproductive health commodities and thereby avoid supply chain gaps – ‘Much of the things that make [the] community come to the facilities is availability of commodities’ (KII DO D1). One commodity particularly highlighted as an incentive for increasing safe delivery at health facilities was a basic *birthing kit* (mama kit) for all women – ‘Those small things which we ask from them at times send [put] them off [from giving birth in a health facility]’ (KII DO D1). Unfortunately, these kits were also in short supply.

Health-system stakeholders emphasised the importance of donor support for short- and medium-term ‘buffer stocks’ (KII DO D1) but also the need ‘to build capacity of not only the district, but also the facilities on how to prioritise and manage commodities’ (KII ZN). Although the situation seemed to have improved, the government’s ability to address the procurement challenges in a sustainable manner remained a concern – ‘If for essential commodities, government still relies heavily on partners, then it is a dangerous situation to be in’ (KII ZN). Donor provisions are mostly associated with particular projects and projects are only funded for limited periods.

#### Financing

Participants expressed concern about the sustainability and maintenance of some of the valued interventions in the MNH programme that required sustained financing. District officials referred to the difference made by support from partners and the implementation of results-based financing (RBF) at health-facility level. Improved performance as demonstrated by the analysis of the collected data meant improved financing through the RBF vehicle, complemented by additional support from partners:

‘RBF funding … has helped us to maintain the hospital. We had a lot of items which are missing … for the management of our cases for maternal-child health. …We tried to buy some … equipment, but where we fell short, UNICEF also came in and it was able to easily procure. …The other factor is just the funding which has improved, which we prioritise’ (KII HF D1).

On the other hand, there were also reports from hospital stakeholders that payment delays through the RBF facility impacted the availability of medicines:

‘We have had babies die, because the mothers cannot afford or they are buying the medicines occasionally. … We have been heavily reliant on … RBF to make sure that we have stock, but the last RBF that came was in quarter two of the last financial year. And even as we talk, we are still demanding money for three quarters, actually four now’ (KII HF D3).

Although health facilities benefited from donor support and RBF, especially where the managers demonstrated good leadership, district-level technical leaders in charge of maternal and child health felt they were underfunded and could not fulfil their leadership and supervisory roles. The absence of a specific budget for MNH at district level also contributed to challenges:

‘There is no budget pointing to maternal and child health. They always say Environmental Health 30% of the PHC [primary health care]. So, which percentage is for maternal health which covers the 80% of the services that are offered? That is a big problem’ (KII DO D3).

### Upgrading neonatal and maternity units and training the health workforce

Quality improvements related to HSS were most visible at health-facility level – ‘Whatever we are doing, we implement at facility level; we pack the knowledge to the facility and it is at that level that we want to see change’ (KII DO D2). Improvements in infrastructure and workforce training were among the interventions particularly valued by healthcare providers.

The establishment, refurbishment and equipping of neonatal care units and high-dependency maternity units for at-risk women at district hospitals were highlights for stakeholders in terms of the reciprocal support between implementing partners and health facilities, with hospitals providing space and partners providing support in the form of equipment, medicines and training.

‘We used to have challenges of monitoring and managing critically ill patients especially those after operation[s]. … And so now we're able to monitor mothers and newborns more effectively’ (KII ZN).

The holistic HSS approach went beyond MNH to include the establishment of WASH facilities, in addition to the provision of neonatal and high-dependency maternity units.

#### Neonatal care units

All informants appreciated the benefits of the neonatal care units with ‘a lot of equipment that is in there’ (KII HF D3) and how these units influenced survival rates:

‘If I'm on gunpoint and have to choose one [intervention] … I think the neonatal care unit has had the most impact as far as I'm concerned’ (KII HF D3).

‘When a baby is born and has some issues, they are handled in that unit to improve on their survival and also help the community because without that facility, it will lead to a lot of referrals, which some of the clients would not afford’ (KII HF D1).

#### High-dependency units

UNICEF, through AVSI, established fully functional and well-equipped high-dependency units – ‘an intermediate between the general ward and the ICU [intensive care unit]’ (KII HF D3) – in all district hospitals in the West Nile region. This idea emerged during the coronavirus pandemic:

‘Because mothers were coming later, there was more need for critical care. And so, making sure that facilities had the space and also had the capacity to not only prevent but also manage [critical care needs] in a timely manner … is an intervention that has arisen in response to this whole COVID situation’ (KII ZN).

Participants described the benefits of the improved maternity wards in term of ‘quality of care of critical patients’ (KII HF D2) and maternal survival, because of the capacity to identify complications – ‘especially [during] the critical monitoring period, the 24 hours, so that if anything goes wrong, it can be detected early enough’ (KII HF D1).

‘Then also the high-dependency units with the space especially for the mothers, which were stretched to help us to greatly cut down on maternal deaths when they are in delivery’ (KII HF D1).

#### *Monitoring of equipmentprovision*

Donations of equipment were not channelled through the district office, but were provided in kind directly to facilities, with donor follow-up on the whereabouts and use of the equipment. This promoted accountability and immediate use of equipment.

‘They [the partner] ask you what equipment you want, and they bring, as opposed to say that now let's give you money and you go and buy. … So, I think one of the best practices [is] their way of following up. … If it is the hospital which has requested [equipment] … they come direct to the hospital store, not to district first. … An officer comes following up to see if they are functional. I think that is a very good best practice’ (KII HF D2).

Maintenance of equipment remained a problem and informants reported that some of the equipment provided was not of the right type or was faulty, examples being oxygen concentrators, incubators and warmers.

#### Other infrastructure improvements

Stakeholders perceived facility-based *WASH improvements* as very important for improving the quality of MNH care and attracting facility-based deliveries. These improvements included rehabilitating health facilities’ water systems and establishing related infrastructure (e.g. tanks, toilets, taps, sinks, bathrooms). This is ‘because sometimes what pulls away [hinders] people from getting some services in the facility is what the facility will look like’ (KII ZN).

The biggest impact of WASH improvements described by participants was the reduction in infections:

‘It has shifted the problem of sepsis … in [our health centre] … which won an award last year in the whole country. … because there's no sepsis, the place is very clean, water is running everywhere’ (KII DO D3).

Another infrastructural deficit that received much appreciated attention was the issue of unreliable *power sources*:

‘Having the standby generator is a beautiful [achievement]. This really stands out. … Our emergency deliveries are no longer at stake [risk] because we have a standby power source which was a very big concern [before]’ (KII DO D2).

On the other hand, there were also fuel challenges to running generators during power outages ‘which the hospital budget cannot offset’ (KII HF D1).

#### Training the health workforce

Establishment of special units was accompanied by thorough training of hospital staff to manage the units and provide comprehensive emergency obstetric and newborn care. Specialists were brought in to train medical officers in order to reduce referrals to regional hospitals. Informants also referred to off-site training and/or on-site mentoring that they felt could have influenced the quality of maternal and newborn care services.

‘When the teams came back [from off-site training], they were able to pass these to other staff. And even when the staff moved on, at least the team they have left behind has picked up from that. And then even the mentorships which have come through, have really improved [the care].’ (KII HF D4)

#### A catch-22 situation

Despite much appreciation for the contributions of partners and the perceived improvement in health outcomes for mothers and babies, the establishment of the special neonatal units and the high-dependency units has also created a dilemma: what is the point of having brand new units but with no change in staffing norms to make it possible to employ additional specialised staff?

‘You know, there is giving you a new unit that was not planned for and you have to squeeze the little human resource that you have to support the community’ (KII HF D3).

‘We recognise that staffing is really, really a challenge and some of the units may become white elephants because of staffing’ (KII ZN).

Provider participants echoed a similar dilemma: what is the point of training staff, amongst others, on the use of equipment, if there is no reliable power source to keep the equipment running?

‘So, that intervention really worked very much because when that capacity was built, we were able to … create a room as a newborn care unit… [but] we still have that massive challenge of power. So, we could not run our incubator, we could not run the warmers which were donated …. Equipment has been there but since we are not connected to the national grid, running that equipment on solar power was difficult. So, we basically just [make] do. We now ask the mothers to do more of kangaroo temperatures [kangaroo mother care]’ (KII HF D4).

### The boda-boda motorcyclist referral system

A key aim of the interventions of the MNH programmes in the West Nile region was to increase utilisation of available services and address two of the delays in MNH care. Village health teams (VHTs) were used to create community awareness of the importance of planning for a health-facility delivery and boda-boda motorcyclists were engaged to transport women to reach the health facility in good time for delivery.

A boda-boda is a commercial motorcycle taxi used to transport people and commodities. A boda-boda referral system was established to transport high-risk pregnant women and newborns with danger signs to health facilities. Selected boda-boda riders from the communities in the catchment areas of health facilities were trained in basic first aid for pregnant women in labour and on how to transport them to a health facility. Participants explained that the boda-boda motorcyclists operated on a voucher system and the women were not required to pay. When a boda-boda operator brought a woman to the facility, he received a voucher. At the end of the month, the boda-boda operators brought their vouchers to the health facility, where they were verified and reconciled with the health facility’s copies. They were then paid by ASVI through Mobile Money, an electronic wallet service for transferring money anywhere in Uganda with a mobile phone.

The free boda-boda referral system was praised by community participants in all the focus groups and individual interviews as having *made a difference* – ‘Money which was [formerly] used for transport has been used to support us in other basic needs’ (IDI Mother D1):

‘Pregnant women and their caretakers are provided transport through the boda-boda services, and this helps mothers a lot, especially in our community where the majority of the mothers are poor and cannot afford transport. These boda-bodas don’t ask for money from the mothers because they are paid by AVSI. Very many women and their children from our community have used the boda riders (FGD VHT D1).’

Boda-boda cyclists perceived the timely referral and availability of transport as ‘making it easy for medical management [of emergencies]’ (IDI BB D3) and saving the lives of mothers and babies. Community stakeholders also referred to the boda-boda system as one of the most significant changes with regard to MNH. Free transport through the boda-boda system meant better access to MNH services – ‘The different service available for the mothers and their babies has also made it easy for us to access the services from within our locality’ (FGD Mother D3). Table 3 contains a narrative by one mother who benefited from actions of the VHT and the boda-boda system.

**Table 3:** Example of the effect of village health teams and the boda-boda referral system on improving maternal and newborn care

I was one of the people who was dodging coming for antenatal care, but there was a woman [VHT] in our village who saw me and discovered that I was pregnant even before my parent realised that I was pregnant. She kept on telling me to come for antenatal care, but for the first time I told her that my pregnancy is too small [early] to go for antenatal care. But she kept on advising me to come, so that I can be helped to have a safe delivery. When she came for the third time, I told her I have started antenatal at this facility [Health Centre B]. On hearing that, she came to the facility, only to be told I did not go. After that she came straight to our home and informed my mother. Hence more pressure was put on me and that is why I responded to their demand.
At the time of my labour the midwives here at Health Centre B tried their level best, but it was not possible to deliver. So they referred me to Hospital X. Then I was like, my mum is not here; she has gone to get money for transport to go to Hospital X. However, I was surprised that even before my mother arrived I saw a boda-boda driver arriving. He started loading my belongings and said that he is taking me to Hospital X. I realised that the midwife called boda-boda to come. Then I told them I was waiting for my mother who had gone to get some money for transport, but they told me the boda-boda would be paid by AVSI. So I was taken to Hospital X without me paying anything and delivered safely from Hospital X.
When I came back from Hospital X and reported at Health Centre B, they also gave me the antenatal [postnatal?] card for free. (FGD Mother D3)

Boda-boda riders elaborated on how the system had *changed their own lives for the better* and earned them ‘respect from community members’ (IDI BB D4). Informants referred to other benefits like an improved standard of living with better nutrition and improved health and the ability to afford school fees, acquire a phone, buy livestock and repair dwellings. Another major benefit mentioned was learning how to manage money prudently and developing ‘a saving culture in my family’ (IDI BB D2):

‘I don’t touch any other money I get from my business … and the money I get normally accumulates and caters for what I planned to buy or acquire which was not the case at the beginning. … I have opened an account with [X] Town Council SACCO, where I am currently saving money I get from AVSI and other sources. I plan to construct a house’ (IDI BB D1).

However, the boda-boda system was not without *challenges*. The most important one for the drivers was delays in payment after verification of the vouchers – it ‘took quite a lot of time to effect payments’ (IDI BB D1). Transporting women back from the health facility was not covered by the voucher system. Individual voices referred to unfulfilled requests for ‘reflector gadgets, gumboots and raincoats’ (IDI BB D1) to make transport safer and ‘to enable us to work in even the rainy season’ (IDI BB D2). Other points included a fixed salary, some form of allowance for riders who carried fewer mothers per month and the provision of motorcycles. It also appeared that the boda-boda system was not functional everywhere at the time of the study – ‘the boda-boda referral ceased’ (IDI VHT D3).

This is how the head of a health centre described both the improvement brought about by the boda-boda cyclists with regard to community delays in care seeking and access and the cyclists’ dilemmas associated with the system:

‘One of the good things is we are able to receive a number of referrals from the community to the facilities. … It has really improved the issues of the deliveries at health-facility level. … Then the challenge is the boda-bodas complain about payment. … their money delays for some time and yet they use their fuel. … So, it has really brought the morale of these boda-boda men a bit down. That's why you see there are some who are not so much active in carrying these referrals to the facility’ (KII HF D2).

## Discussion

The MNH quality-improvement programme in the West Nile region adopted a holistic, HSS approach based on evidence of effective interventions applied elsewhere [18] and addressed health-system gaps at district, health-facility and community level. The findings illustrate how different health programmes and building blocks do not work separately in silos, but instead work in conjunction to improve service delivery [19, 20]. This may also explain stakeholder perceptions of that interventions were equally important, with the inclusion of broader interventions that impacted on the delivery of the MNH programme (e.g. WASH, use of data and RBF). The interventions that stakeholders valued were also a fulfilment of the policy prescriptions for a high-impact maternal and newborn care package in Uganda [21].

The data-driven approach at district and facility level set the scene for measuring maternal and newborn health outcomes and monitoring the implementation of MNH interventions at the facility and community levels. Facility-based activities that stakeholders at district and facility levels perceived to have improved the quality, uptake and utilisation of MNH services and maternal and newborn health outcomes included the refurbishment of neonatal and maternity units (including upgrades of WASH facilities and the provision of additional power sources) and the upskilling of health managers and frontline healthcare providers through training, on-site mentoring and other capacity-building activities. Community-based interventions perceived to increase maternal and newborn survival included the boda-boda-referral transport system.

The Ugandan government is committed to the use of ‘data for action’. This approach is included not only in the Every Newborn Action Plan (ENAP) [22] and the Ending Preventable Maternal Mortality (EPMM) [23] documents, but also in the survive, thrive and transform agenda ‘to end preventable deaths (survive); ensure health and well-being (thrive); and change how small and sick newborns are cared for (transform)’ (page 91) [24]. The description in our paper focused largely on interventions aimed at transforming the health system to help mothers and their newborns to survive and thrive through quality care. The WHO-UNICEF Survive and Thrive report suggests indicators for the different levels of the health system. National tracking data should include indicators for impact, coverage, service readiness, human resources, equipment and drugs. At the district level data coverage indicators should be complemented with more detailed indicators of service readiness, equipment and drugs. Facility-level management should be responsible for quality improvement process data [24].

The upgrading of facility infrastructure is an intervention that appears in many donor-supported MNH programmes to improve the provision of quality maternal and newborn care. Planning of and investment in hospital infrastructure and capacity building of health providers for small and sick newborn care are currently high on the health agenda in low- and middle-income countries (LMICs) [25] and are also part of the West Nile MNH programme. Two of the meso-level subtype interventions identified by a systematic review of quality improvement initiatives for hospitalised sick and small newborns in LMICs were strengthening facility infrastructure and in-service training (the most featured subtype) [25]. The challenges emanating from infrastructural improvements and human resource challenges mentioned in our study are commonly reported in other studies [26, 27]. One issue that strongly emanated from our study is that there should be a comprehensive strategy for addressing human resources at national level.

Globally governments and development partners are continuously considering innovative ways to reduce the delays that can occur between a pregnant woman and her family’s decision to seek facility-based delivery care and her arrival at a health facility. This discussion usually includes available transport options. Boda-boda transport is common in countries like Uganda, Kenya and Tanzania and also elsewhere in the world, where it may be known by other names. One Ugandan study reported on training boda-boda riders for transporting emergency medical cases in general [28]. Two further studies showed an increase in facility-based antenatal and/or delivery care. A non-randomised controlled trial in the Busoga region of Uganda used monetary and non-monetary incentives for mothers, midwives, VHTs and boda-boda riders and showed both an increase in the use of boda-boda transport by expectant women to reach a facility for delivery and a decrease in response time from trained boda-boda riders [29]. A quasi-experimental pilot study in Eastern Uganda using supply- and demand-side incentives – including vouchers for transport – showed a marked increase in facility-based antenatal and delivery care, which again dropped off at the end of the pilot [30]. In addition, economic spill-over effects on the family and dependants – similar to the findings in our study – were also reported [31]. The study also reported concerns with regard to sustainability and cost-effectiveness [30]. None of these studies reported on the impact of this intervention.

The three-delay model has been widely used in LMICs to identify barriers to access to obstetric care with a view to designing evidence-based interventions to improve care and service delivery [32]. Uganda was the first country to expand the application of the three-delay model to explore the identification of causes of newborn mortality [33]. Although there are many references to the three-delay model in Ugandan policy documents and instruments, according to Mukuru *et al.*, the piecemeal additions to policy instruments and the lack of coherence and consistency have resulted in the persistence of the three delays [34]. In our study, stakeholders referred to challenges in the continuity of delivering donor-supported interventions aimed at addressing the three delays and raised questions about sustainability without increased contributions from government. This was mentioned particularly with reference to resources for essential medications and fuel, the staffing of upgraded maternal and newborn units, and the boda-boda transport system.

Stakeholders had positive perceptions of the MNH interventions in the West Nile region, but also referred to challenges, gaps and bottlenecks. This suggests that the design of interventions based on the health-systems building blocks framework and the three-delay model may not be able to capture all the dynamic and complex processes and outcomes of an interlinked system and the broader community determinants of health-seeking behaviour [35, 36]. A case in point that was emphasised in our study was the upgrading of infrastructure and the provision of equipment, but also the non-availability of more healthcare providers, incompatibilities between equipment and the power supply, and the lack of fuel for generators. Cessation or deterioration of certain interventions and delays in payment could well demoralise frontline healthcare workers and community providers in the long run. Furthermore, interventions should transcend mere technology and the provision of a reliable power supply for special care units to include a person-centred enabling environment with privacy, positive healthcare provider communication, and positive facility leadership structures as the basis for sustained improvement in respectful and dignified maternal and newborn care [37].

While policies and strategies in Uganda are well formulated at the national level, stakeholders in our study alluded to having limited resources for the operationalisation of MNH policies at the district, health-facility and community levels. There were indications that at a national level some of the more general priorities had not been addressed sufficiently and general gaps in staffing norms and insufficient funding for district health offices were perceived to impact on the provision of MNH services. It is common for insufficient funding to be underrated at a central level, from which only limited funding is provided, with strings attached. Fiscal decentralisation of sufficient resources and district management has been challenging in several settings [19, 38].

Furthermore, donor support should not be seen as a panacea for all health-system problems. Sociologists have argued that the discourse of sustainability among donors and non-governmental organisations actually contributes to the undermining of HSS projects. This is based on the so-called ‘sustainability doctrine’ which favours short-term funding streams for targeted interventions instead of addressing the long-term needs of local populations [39, 40]. Sustainability was also raised in our study with regard to the maintenance of WASH facilities, the financing of essential commodities and the appointment and retention of special-skills staff for the newly established maternal and newborn units.

It has been argued that HSS should consider establishing resilient systems [41]. Most sources included in a scoping review by Fridel *et al.* referred to the characteristics of health-system resilience in terms of adaptation, maintenance, absorption, learning, transformation, and withstanding and responding to shocks [42]. These characteristics have not been explored in our study and merit further attention.

### Methodological considerations and limitations

The study was conducted in only four districts in one region of Uganda. A purposive sample covered four diverse districts, different levels of the health system and provider and beneficiary stakeholders to allow for maximum variation. Our study did not elicit the perspectives of community leaders, however.

The use of snowballing through the hierarchies of the health system may have introduced bias because potential participants were known to the level above, so power imbalances may have come into play. The fact that study participants included members involved in the implementation of the quality-improvement programme in the West Nile region may also have created social desirability bias in participant descriptions of the interventions. To reduce potential bias, investigators included probes on challenges experienced in the implementation of the MNH programme in the KIIs and compared perspectives across the different districts and health-system levels. Although the findings may not be generalizable because district contexts and leaderships vary and each district has its own configuration of partners, other districts and countries may find many of the findings transferable because of the universality of the interventions included in the programme. Table 4 provides a list of recommendations for consideration in planning the way forward for the MNH programme.

**Table 4:** Recommendations for the way forward for the MNH programme in the West Nile region

**Data management and use**
• Continue sensitisation on the importance of using data for action and support training and refreshers on data use to enlarge the competent staff pool at district and health-facility level.
• Strengthen the data collection systems through well-defined indicators and appropriate data collection tools, including electronic medical records and support for the infrastructure required for such innovations.
• Improve the use of data reviews, specifically at health-facility level and respond to the identified bottlenecks and needs.
**Financing**
• Advocate for and obtain more secure investments in the MNH programme for longer time periods.
• Reconsider how funding for MNH services is allocated and how it is managed at district level to minimise the mismatch between national and district expectations and perceptions.
• Create budget lines for the allocation of funds for MNH at district level.
• Review the payment system for boda-boda riders to ensure timeous payment of vouchers to ensure continuity of this essential community referral system.
**Health workforce**
• Lobby the Ministry of Health to review the staffing structure remuneration for all health facility levels in the country and revise staffing levels for critical cadres (e.g. doctors, anaesthetic officers and midwives).
• Include newly recruited medical officers in capacity building programmes for MNH to enhance their skills in the provision of CEmONC and small and sick newborn care.
• Ensure the continuation of maternal and newborn care training for new staff and refreshers for existing staff.
**Supply chain**
• Prioritise lifesaving commodities for women and children recommended by United Nations agencies.
• Strengthen the logistics and supply chain management systems to address the issues of late deliveries of supplies, discrepancies between ordered and delivered supplies and delivery of drugs close to their expiry date.
• Lobby the national medical stores to establish a system to monitor the packaging of supplies and equipment and to review delivery schedules.
**Infrastructure and equipment **
• Include the construction and maintenance of maternity and newborn units in hospital designs and budgets.
• Explore innovative ways to ensure the maintenance of the maternity and newborn units, including the WASH facilities.

*MNH* maternal and newborn health, *CEmONC* comprehensive emergency obstetrics and newborn care, *WASH* water, sanitation and hygiene

## Conclusion

The MNH programme in the West Nile region adopted a holistic and system-wide approach to addressing the key bottlenecks in the planning, delivery, and monitoring of quality MNH services and was largely shaped by a conducive policy environment and outstanding donor support. Stakeholders at the various levels of the health system expressed appreciation for the most important innovations included in the programme to improve quality, to provide the needed facilities for safe delivery and to reduce all three sources of delay. Although there was a general view across the board that the interventions had the potential to improve newborn and maternal health outcomes, the implementation of HSS initiatives needs to be well planned and can be suboptimal if resource constraints do not allow for appropriate alignment between the health-systems building blocks and the different levels of care. Since the funding was donor-driven, questions about government ownership and sustainability in the context of limited resources remain.

## Disclaimer

The views are those of the authors and do not reflect the official position of the United Nations Children’s Fund.

## Supplementary Information

**Additional file 1.** 

## Data Availability

The datasets generated and/or analysed during the current study are not publicly available because it was a qualitative study but are available from the corresponding author on reasonable request.

## Abbreviations

HSS: Health-systems strengthening
FGD: Focus group discussion
IDI: In-depth individual interview
KII: Key informant interview
LMICs: Low- and middle-income countries
MMR: Maternal mortality ratio
MNH: Maternal and newborn health
RBF: Results-based financing
RMNCAH: Reproductive, Maternal, Newborn, Child and Adolescent Health
UNICEF: United Nations Children’s Fund
VHT: Village health team
WASH: Water, sanitation and hygiene
WHO: World Health Organization
