# Supplementary material for: Perspectives of stakeholders regarding the value of maternal and newborn health interventions and practices supported by UNICEF and other partners in the West Nile region of Uganda: a qualitative study
Source: BMC Health Serv Res. 2023 May 11;23:473. doi: 10.1186/s12913-023-09480-x (PMC10172057; doi:10.1186/s12913-023-09480-x)
Supplement: Supplementary file 1 — Additional file 1. [file 12913_2023_9480_MOESM1_ESM.pdf]

NVivo nodes – key informant interviews

- PARTNERS
  - UNICEF contributions and approach
  - AVSI Contributions
  - Other partners
- GENERAL (CHALLENGES, ACHIEVEMENTS, LESSONS)
  - Challenges – general
  - Achievements and dilemmas - general
  - Lessons – general
- CONTINUUM OF CARE
  - Antenatal care
  - HIV (& PMTCT)
  - Deliveries facility
  - KMC
  - Postnatal care (incl Immunisation)
  - Nutrition (& breastfeeding)
  - Family planning
- COMMUNITY (DEMAND SIDE)
  - Cultural beliefs
  - Male involvement
  - Gender-based violence
  - Community delays (1st delay)
  - Health-seeking behaviour
  - Community dialogues & integrated outreach
  - Health education
  - Refugees
  - Village Health Teams (& family practice mothers) – expert mothers, peer mothers
- REFERRAL (2ND DELAY)
  - Challenges with referral (& solutions)
  - Ambulance system and boda bodas (and voucher system)
- HEALTH SYSTEMS STRENGTHENING
  - MNH programme
  - Health system
  - Ministry of Health (& government)
  - DHT strengthening & league tables
  - District achievements
  - Leadership
  - Quality improvement & QoC
  - Lessons Passion & Respectful Care
  - Client satisfaction
  - Sustainability
- INFRASTRUCTURE & EQUIPMENT (SUPPLY SIDE)
  - Water system & sanitation (incl WASH & IPC)
  - Blood bank
  - Maternity waiting home
  - Renovations wards
- COMMODITIES, SUPPLIES AND MEDICINES (SUPPLY SIDE) (3RD DELAY)
  - Commodities & medicines (& MamaKit)
  - Supplies
- HUMAN RESOURCES (3RD DELAY)
  - Human resources (achievements)
  - Challenges & lessons HR
  - Challenges HR
- TRAINING, OUTREACH AND MENTORSHIP
  - Training (skills & gaps) (3rd delay & QoC)
  - Training (& mentorship) (capacity building)
  - Support supervision (outreach & mentorship)
- HMIS
  - Audit, mapping & data use
  - Indicators & MPDSR
  - Challenges stillbirths
  - Performance reviews
  - FamilyConnect & digital health system
- FINANCING
  - Financing (challenges and models)
  - Rewards as enabler
- COVID
- QUOTABLE QUOTES
